# Structural equation modeling to identify social and physical risk indicators of early childhood caries in suburban Nigerian population

**DOI:** 10.3389/froh.2026.1846301

**Published:** 2026-07-20

**Authors:** Farzana Haque, Fardous Hasan, Mohammad R. Khami, Roberto Ariel Abeldaño Zuñiga, Maha El Tantawi, Simin Z. Mohebbi, Moréniké Oluwátóyìn Foláyan

**Affiliations:** 1 Department of Global Public Health and Primary Care, University of Bergen, Bergen, Norway; 2 Department of Clinical Dentistry, University of Bergen, Bergen, Norway; 3 Haraldsplass Diakonale Sykehus, Bergen, Norway; 4 Department of Information Science and Media Studies, University of Bergen, Bergen, Norway; 5 Research Center for Caries Prevention, Dentistry Research Institute, Tehran University of Medical Sciences, Tehran, Iran; 6 Community Oral Health Department, School of Dentistry, Tehran University of Medical Sciences, Tehran, Iran; 7 Centre for Social Data Science, Faculty of Social Sciences, University of Helsinki, Helsinki, Finland; 8 Department of Pediatric Dentistry and Dental Public Health, Faculty of Dentistry, Alexandria University, Alexandria, Egypt; 9 Department of Child Dental Health, Obafemi Awolowo University, Ile-Ife, Nigeria

**Keywords:** biopsychosocial model, commission on social determinants of health, oral hygiene, public health interventions, social determinants of health

## Abstract

**Background:**

Early Childhood Caries (ECC) is a growing public health problem in low and middle-income countries like Nigeria. This study aimed to identify the social and physical risk indicators of ECC among children in the Ile-Ife Central Local Government Area of Nigeria.

**Methods:**

A cross-sectional study was conducted among 895 mother-child dyads aged 0–60 months between December 2024 and January 2025. The outcome variable was ECC experience. The latent variable**s** were socioeconomic status (SES), maternal oral health knowledge (MOHK), oral hygiene status (OHS), and household dietary sugar exposure (HDSE). Child age and maternal time availability for breastfeeding were included as observed covariates. Diagonally Weighted Least Squares Structural Equation Modelling (DWLS-SEM) was utilized to fit the model, with bootstrap estimation (1,000 resamples) for indirect and total effects.

**Results:**

Among 895 mother-child dyads, 66 children (7.4%) had ECC and 1.5% had PUFA ≥ 1. The model demonstrated good fit (CFI = 0.959, TLI = 0.952, RMSEA = 0.045). There was a significant positive association between SES and maternal knowledge (*β* =  + 0.47, *p* < 0.001), and negative associations between SES and oral hygiene status (*β* = −0.17, *p* = 0.004) and ECC (*β* = −0.15, *p* = 0.034). Oral hygiene status showed the strongest direct association with ECC (*β* =  + 0.32, *p* = 0.008). Child age had a significant indirect effect on ECC via oral hygiene (*β* =  + 0.10) but no significant direct effect. Higher maternal knowledge was paradoxically associated with poorer oral hygiene (*β* =  + 0.13, *p* = 0.010). Limited maternal time for breastfeeding was positively associated with ECC (*β* =  + 0.10, *p* = 0.020). Dietary sugar exposure showed a small but marginal significant model-level association (*β* = +0.13, *p* = 0.08). The total effect of SES on ECC (*β* = −0.19) exceeded its direct effect, with oral hygiene mediating the largest indirect pathway.

**Conclusion:**

The findings highlight that ECC in this population is primarily structured by socioeconomic position rather than knowledge alone, underscoring the need for interventions addressing structural inequalities and resource constraints alongside behavior change strategies.

## Introduction

Early Childhood Caries (ECC) is a major global public health concern, with a prevalence approximately 7,550 per 100,000 children with deciduous teeth in 2021, with an age-standardized DALY rate of 2.89 per 100,000 population (1). Defined as decayed, missing, or filled tooth surfaces in primary teeth of children under six (2), ECC extends beyond oral health, being associated with pain and discomfort that impair eating and contribute to poor nutrition and malnutrition (3–5). Dental pain also disrupts sleep, affect cognitive development and emotional regulation (6, 7), while tooth loss impairs speech (8, 9), communication, and social interactions (10). ECC increases the risk of future dental caries in permanent teeth (11, 12) and orthodontic problems (13), and may perpetuate poor oral health into adulthood (14). Collectively, these effects reduce quality of life (4), self-esteem (15, 16), academic performance (17, 18), school attendance (19), and participation in social activities (20).

Economically, ECC places a heavy burden on families and healthcare systems. Treatment of advanced cases, particularly those requiring hospital-based care under general anaesthesia, is prohibitively costly (21, 22), especially for low-income households. Additional indirect costs such as transportation (23), lost wages from caregiving (24), and reduced productivity (25) further strain family resources (26). Healthcare systems also face significant expenses from emergency treatments and managing long-term complications of untreated ECC (27).

Despite being preventable, ECC remains highly prevalent in low- and middle-income countries with limited access to oral healthcare and preventive services (28), and untreated cases in Africa increased by 93.9% between 1990 and 2021 (1). Multiple risk factors are associated with ECC (29), spanning individual, social, and environmental determinants (30). In Nigeria, where the prevalence of ECC is 17% (31), identified risk factors include older age, male gender, inconsistent socioeconomic associations, limited dental service utilization, and residential environments that foster harmful oral practices (32). Additional indicators include maternal factors (poor oral health knowledge, maternal dental caries, spontaneous membrane rupture during delivery, and high parenting stress), elevated levels of Streptococcus mutans and Streptococcus sobrinus, enamel defects, and low CD4 counts. They also include children's oral health behaviors such as poor hygiene, once-daily toothbrushing, glycerin uses for mouth cleaning, frequent sugary snack consumption, prolonged or night-time bottle feeding, breastfeeding beyond 12 months, and malnutrition (32).

Despite this breadth of identified risk factors, the mechanisms through which structural, and cognitive determinants converge and where the pathway from knowledge to behavior to clinical outcomes breaks down remain poorly characterized in the sub-Saharan African context. Prior Nigerian ECC studies have largely used univariate or multivariate regression frameworks (33) that cannot estimate the direct and indirect pathways linking socioeconomic position to distal oral health outcomes (34). To better capture these interrelationships, structural equation modeling (SEM) provides a more suitable analytical framework. However, where SEM has been applied in oral health research, maximum likelihood (ML) estimation has predominated, which relies on the assumption of multivariate normality (35). This assumption is often violated in community-based surveys where indicators are measured using ordinal scales or binary response formats (36). Consequently, conventional SEM approaches may yield biased or less reliable estimates in such contexts (37). To our knowledge, no published study has applied a Diagonally Weighted Least Squares (DWLS) based SEM (DWLS-SEM) framework to model the socioeconomic, cognitive, dietary, and clinical determinants of ECC and decompose their direct and indirect effects in a Nigerian population.

**Figure 1:**
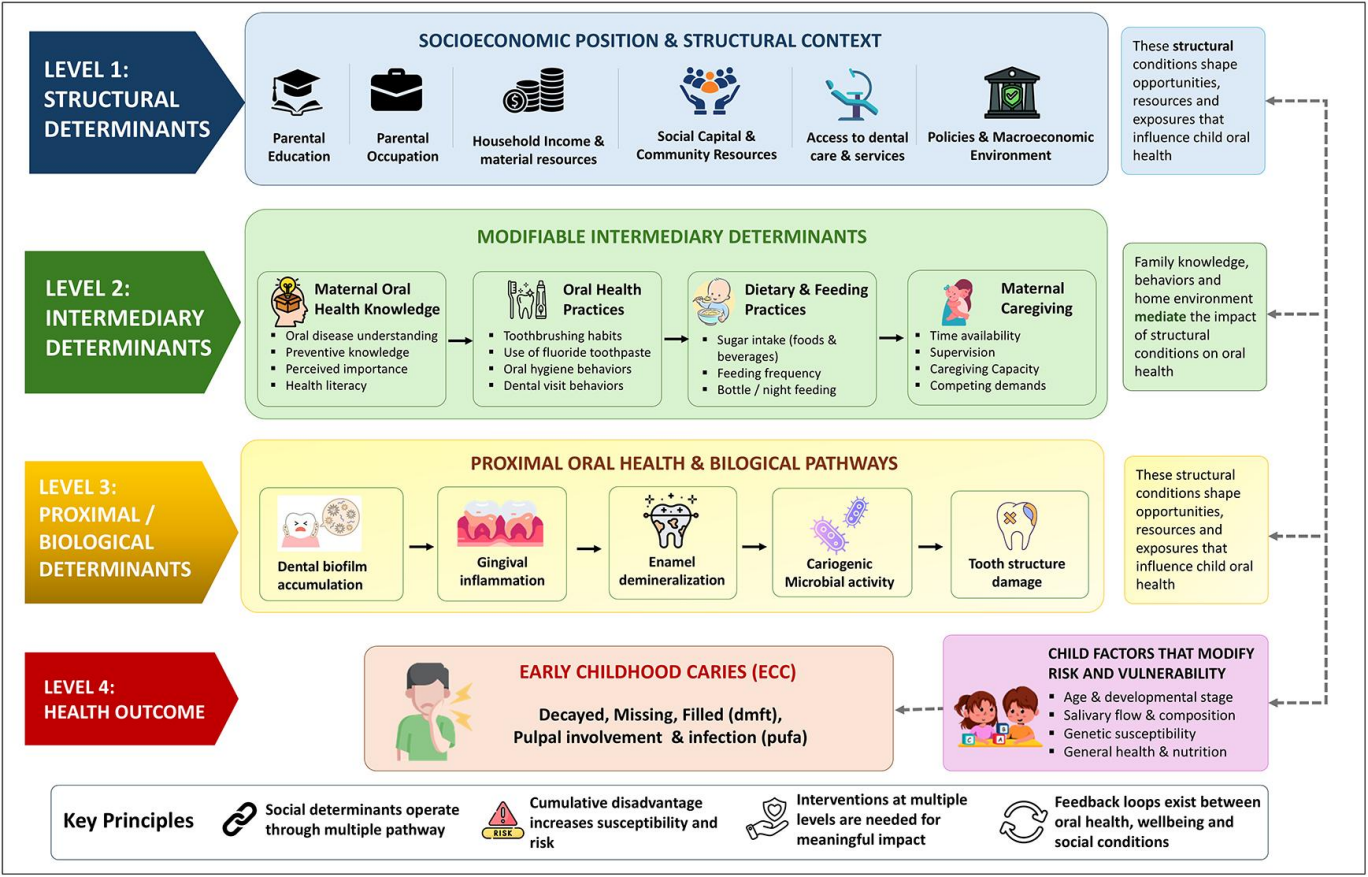
Conceptual framework of structural, intermediary, and clinical determinants of early childhood caries based on the WHO Commission on social determinants of health.

To address these gaps, this study applies SEM estimated using DWLS, which accommodates non-normally distributed and ordinal data commonly observed in community-based oral health surveys (38), and estimates direct and indirect pathways among multiple determinants. The model is guided by the WHO Commission on Social Determinants of Health (CSDH) framework (39) and which positions socioeconomic status as a structural upstream determinant that shapes intermediary factors including health knowledge, dietary practices, maternal monitoring capacity, and clinical oral hygiene status, which in turn produce differential health outcomes (40, 41). Within this framework, parental education and occupation define structural socioeconomic position; parental oral health knowledge, dietary sugar exposure, and maternal time availability operate as intermediary determinants; oral hygiene status serves as the proximal biological mediator; and ECC experience constitutes the distal health outcome. Importantly, the model tests whether structural constraints are sufficiently severe that knowledge does not translate into measurable hygiene improvement—a hypothesis consistent with CSDH's emphasis on material conditions as dominant over cognitive factors in resource-constrained settings (39). Figure 1 presents the hypothesized pathway model. The model specifies four hierarchical levels: structural socioeconomic position, parental oral health knowledge and intermediary behavioral determinants (dietary sugar exposure and maternal monitoring capacity), oral hygiene status as the proximal clinical mediator, and ECC experience as the distal outcome.

This study, therefore, aimed to assess the direct and indirect associations between parental socioeconomic position, parental oral health knowledge, dietary sugar exposure, “maternal time for breastfeeding, and oral hygiene status with ECC experience among children aged 0–60 months in Ile-Ife Central Local Government, Nigeria, using a CSDH-grounded DWLS-SEM.

## Methods

### Ethical approval and informed consent

The study protocol was reviewed and approved independently by the Tehran University of Medical Sciences, Tehran, Iran (reference: IR.TUMS.DENTISTRY.REC.1402.023) and the Institute of Public Health Research Ethics Committee of Obafemi Awolowo University, Ile-Ife, Nigeria (reference: IPH/OAU/12/2742). Written informed consent was obtained from the mother or legal guardian of each enrolled child prior to any data collection. Participation was entirely voluntary, no compensation was offered, and all records were de-identified before analysis; names and residential addresses were not recorded.

### Study design and population

A community-based cross-sectional study was conducted among children aged 0–60 months residing in Ile-Ife, Osun State, Nigeria. The study population comprised mother-child dyads in which the child was living with the biological mother or a legal guardian at the time of enumeration. Eligibility required only the written consent of the parent or guardian and the child's presence at home during the survey period; no medical or sociodemographic exclusion criteria were applied.

### Study location

The dataset included geographic coordinates (latitude and longitude) for each child. A geospatial clustering approach was applied using the Nominatim geocoder, which converted these coordinates into structured address information (42). Based on this process, the dataset was classified into three geographic clusters: “Ife Central”, “Ife East”, and “Ife North”. This clustering method allowed for the systematic grouping of similar geographic points, simplifying the analysis of spatial patterns. Analysis was restricted to the Ife Central cluster (*n* = 915 prior to data cleaning) to enhance within-sample environmental and socioeconomic homogeneity while retaining sufficient statistical power (43).

### Sample size

Using the Cochran formula (44), the minimum number of children to be screened was calculated at 969, based on an anticipated ECC prevalence of 4.3% (45), a 5% margin of error, and a 95% confidence level. A total of 969 children were enrolled across all enumeration areas. Following geospatial delineation, analysis was restricted to the Ife Central cluster (*n* = 915), from which 895 mother-child dyads with complete data on all model variables constituted the final analytic sample. This exceeds the recommended minimum of 500 observations for SEM with more than seven constructs (46).

### Sampling procedure

The random sampling procedure for this study was modelled after previous ECC household surveys. In Stage 1, the Ile-Ife Central Local Government was chosen for its suitability as a long-term ECC surveillance site, having hosted similar surveys in 2014 (47) and 2020 (45), making this the third wave of data collection. In Stage 2, 10% of the 700 enumeration areas from the 2006 National Population Commission Census were randomly selected by balloting, a percentage deemed representative for household surveys (48). Stage 3 involved identifying eligible households within these areas, with every other household on each street considered for recruiting a mother-child dyad. In Stage 4, respondents were selected for interviews and clinical examinations, ensuring only one dyad per household participated. Recruitment continued in each enumeration area until the enlisted enumeration areas were covered.

### Data collection and clinical examination

Data collection took place between December 2024 and January 2025 through structured interviewer-administered questionnaires and standardized dental examinations. Twenty data collectors underwent pre-survey training covering the study protocol, research ethics, and interviewing techniques. A pilot study identified logistical challenges in questionnaire administration. The skip order and wording of selected items were subsequently refined to optimize response accuracy. The questionnaire collected data across four thematic domains: sociodemographic characteristics of the child, mother, and father; maternal oral health behaviors and knowledge; child oral hygiene practices and supervision; and infant and child dietary and feeding practices.

Dental caries experience was assessed by nine calibrated dentists using the dmft index according to WHO criteria (49). Inter-examiner reliability was evaluated by Fleiss' kappa prior to the main data collection; the initial kappa of 0.62 indicated moderate-to-substantial agreement but fell below the pre-specified threshold of 0.80.

### Statistical analysis

#### Pre-processing and measurement framework

Prior to DWLS-SEM, the dataset underwent systematic pre-processing. As all indicators were ordinal or binary, Spearman's *ρ* was selected as the inter-item association metric. To ensure sufficient construct homogeneity, the primary criterion required inter-item correlations to fall within the range of 0.15–0.50 (50). Cronbach's *α* ≥ 0.50 was used as a secondary internal consistency check. This lower-than-conventional threshold (0.70) (51) was chosen because *α* has a downward bias in scales with few items (52, 53). For two-item constructs, the criterion was bivariate Spearman *ρ* ≥ 0.30 using the Spearman-Brown reliability estimation (54). *post-hoc* convergent validity was assessed using composite reliability (CR) and average variance extracted (AVE). When AVE fell below 0.50, the construct was retained provided CR was satisfactory (55).

#### Latent constructs

Five constructs met the criteria for reflective specification and were retained in the final model.

##### Socioeconomic Status ($SES-\;\eta_{SES}$)

SES measured by four observed indicators: father's education ($\chi_{1}$), mother's education ($\chi_{2}$), father's occupation ($\chi_{3}$), and mother's occupation ($\chi_{4}$). Maternal education was categorized on a four-point ordinal scale: 0 = no formal education, 1 = primary education, 2 = secondary education, and 3 = tertiary education. Maternal occupation was classified into four hierarchical categories: 1 = unemployed/student, 2 = unskilled, 3 = skilled/self-employed, and 4 = civil servant. Higher SES values reflect more favorable socioeconomic conditions.

##### Maternal oral health knowledge (**MOHK—**$\eta_{MOHK}$)

MOHK was constructed by six observed indicators assessing knowledge in preventing dental caries: Effectiveness of water fluoridation ($\chi_{5}$), Effectiveness of fluoride toothpaste ($\chi_{6}$), Fluoride is more important than brushing ($\chi_{7}$), twice-daily brushing ($\chi_{8}$), importance of regular dental visits ($\chi_{9}$) and Rinsing reduces fluoride effect ($\chi_{10}$). All items were rated on a five-point Likert scale with the following response categories: 1 = do not know, 2 = strongly disagree, 3 = disagree/uncertain, 4 = agree, and 5 = strongly agree, such that higher values represent better knowledge. Lower values therefore reflect poor knowledge characterized by uncertainty, disagreement with evidence-based preventive measures, or lack of awareness regarding fluoride effectiveness, brushing practices, and the importance of regular dental visits.

##### Household dietary sugar exposure (HDSE—$\eta_{HDSE}$)

HDSE derived from three items: child refined carbohydrate intake between meals ($\chi_{11}$), frequency of sugar-containing food consumption ($\chi_{12}$), and frequency of sugar-containing drink consumption ($\chi_{13}$). All items were rated on a three-point ordinal scale: 1 = rarely/never, 2 = sometimes, and 3 = often/very often, with higher scores representing more frequent sugar exposure.

##### Oral hygiene Status (**OHS—**$\eta_{OHS}$)

OHS derived from three clinical measures: oral hygiene index ($\chi_{OHI}$), the gingival index ($\chi_{GI}$), and plaque index ($\chi_{PI}$). Higher scores indicate worse oral hygiene.

##### Early childhood caries (**ECC**—$\eta_{ECC}$)

ECC was indicated by dmft score ($\chi_{dmft}$) and pufa score ($\chi_{pufa}$). Both indicators reflect the same underlying caries process. Bivariate *ρ* ($\chi_{dmft}$, $\chi_{pufa}$) = 0.311 (*p* < 0.001) meets the two-item threshold.

#### Observed variables

Observed variables were included in the model as directly measured indicators without latent specification. Child Age ($\chi_{\mathrm{age}}$) was recorded in months (range: 0–60) as a continuous covariate for cumulative ECC exposure. Maternal time for breastfeeding ($\chi_{bfeeding}$**)** was measured on a binary scale: 1 = has adequate time, 2 = insufficient time. Higher values indicate greater time constraints limiting maternal time available for breastfeeding.

#### Structural equation model

Following variable operationalization, DWLS-SEM was employed to evaluate the hypothesized relationships among the reflective latent constructs, observed variables, and the ECC. The latent variables in the final model were $\eta_{SES}$*,* $\eta_{MOHK}$**,** $\eta_{HDSE}$**,** $\eta_{OHS}$**,** and $\eta_{ECC}$. Two individual variables ($\chi_{age}$, $\chi_{bfeeding}$**)** were included as observed variables.

##### Measurement model

The reflective latent constructs were defined as linear combinations of their observed indicators:

$$\eta_{SES}=\;\lambda_{1}.\chi_{1}+\lambda_{2}.\chi_{2}+\lambda_{3}.\chi_{3}+\lambda_{4}.\chi_{4}+\epsilon_{SES}$$

$$\eta_{MOHK}=\;\lambda_{5}.\chi_{5}+\lambda_{6}.\chi_{6}+\lambda_{7}.\chi_{7}+\lambda_{8}.\chi_{8}+\lambda_{9}.\chi_{9}+\lambda_{10}.\chi_{10}+\epsilon_{MOHK}$$

$$\eta_{HDSE}=\;\lambda_{11}.\chi_{11}+\lambda_{12}.\chi_{12}+\lambda_{13}.\chi_{13}+\epsilon_{HDSE}$$

$$\eta_{OHS}=\;\lambda_{14}.\chi_{OHI}+\lambda_{15}.\chi_{GI}+\lambda_{16}.\chi_{PI}+\epsilon_{OHS}$$

$$\eta_{ECC}=\;\lambda_{17}.\chi_{dmft}+\lambda_{18}.\chi_{pufa}+\epsilon_{ECC}$$

where $\lambda_{i}$ represent factor loadings, $\eta_{i}$ the latent construct and $\epsilon_{i}$ represent measurement error terms. For model identification, one factor loading per latent variable was fixed to 1.0.

##### Structural model

Three structural equations were specified through a series of regression equations, with β representing path coefficients and δ representing structural disturbance terms:

The model equations are:

$$\eta_{MOHK}=\beta_{1}.\eta_{SES}+\delta_{MOHK}$$

$$\eta_{OHS}=\beta_{2}.\eta_{MOHK}+\beta_{3}.\eta_{SES}+\beta_{4}.\chi_{age}+\delta_{OHS}$$

$$\eta_{ECC}=\beta_{5}.\eta_{OHS}+\beta_{6}.\eta_{SES}+\beta_{7}.\chi_{age}+\beta_{8}.\chi_{bfeeding}+\beta_{9}.\eta_{HDSE}+\delta_{ECC}$$

##### Covariance structure

Covariances among predictor variables were specified as $\phi_{i}$ parameters, representing non-causal associations not captured by directional paths.

$Cov(\eta_{SES},\;\chi_{age})=\phi_{1}$**;** $Cov(\eta_{SES},\;\chi_{breastfeeding})=\phi_{2}$**;** $Cov(\eta_{SES},\;\eta_{HDSE})=\phi_{3}$**;**

#### Estimation and inference

The DWLS-SEM fit was evaluated using CFI, TLI, and RMSEA as primary indices, with acceptable thresholds of CFI/TLI ≥ 0.90 and RMSEA ≤ 0.08 (56); the *χ*^2^/df ratio was reported but not treated as a primary criterion given its sensitivity to large samples (57). To assess parameter stability, non-parametric bootstrapping with 1,000 resamples was performed with confidence interval. All analyses were conducted in Python 3.11 using semopy 2.3, scikit-learn 1.4, scipy 1.12, pandas 2.2, and numpy 1.26.

## Results

### Sample characteristics

A total of 895 mother-child dyads were included in the analysis (Table 1). Children had a mean age of 31.1 months (SD = 18.8), with the largest age group being 48–60 months (34.1%). Slightly more than half of the children were female (53.1%). Regarding socioeconomic status, the sample was predominantly middle-tier. Most mothers (61.6%) and fathers (58.3%) completed senior secondary education, with an additional 26.0% of mothers and 33.9% of fathers attaining tertiary education. Similarly, the majority of mothers (60.4%) and fathers (72.2%) were engaged in skilled or self-employed occupations. Maternal oral health knowledge regarding ECC prevention was limited.

**Table 1:** Descriptive characteristics of the study sample (*N* = 895).

Characteristic	Category/Statistic	*n*	(%)
Child Characteristics			
Age (months)	Mean (SD)—31.1 (18.8)		
	< 12 months (infant)	88	9.8%
	12–23 months	179	20.0%
	24–35 months	175	19.6%
	36–47 months	148	16.5%
	48–60 months	305	34.1%
Gender	Male	420	46.9%
	Female	475	53.1%
Socioeconomic Status (SES)			
Mother's education	Primary	33	3.7%
	Junior secondary	78	8.7%
	Senior secondary	551	61.6%
	Tertiary	233	26.0%
Mother's occupation	Unemployed/student	94	10.5%
	Unskilled/informal	218	24.4%
	Skilled/self-employed	541	60.4%
	Professional/civil service	42	4.7%
Father's education	Primary	32	3.6%
	Junior secondary	38	4.2%
	Senior secondary	522	58.3%
	Tertiary	303	33.9%
Father's occupation	Unemployed/student	20	2.2%
	Unskilled/informal	141	15.8%
	Skilled/self-employed	646	72.2%
	Professional/civil service	88	9.8%
Maternal Oral Health Knowledge (MOHK) in Preventing Early Childhood Caries			
Effectiveness of water fluoridation	Don't know	692	77.3%
	Strongly disagree	4	0.4%
	Disagree	9	1.0%
	Agree	113	12.6%
	Strongly agree	77	8.6%
Effectiveness of fluoride toothpaste	Don't know	570	63.7%
	Strongly disagree	5	0.6%
	Disagree	10	1.1%
	Agree	201	22.5%
	Strongly agree	109	12.2%
Fluoride is more important than brushing	Don't know	599	66.9%
	Strongly disagree	8	0.9%
	Disagree	19	2.1%
	Agree	173	19.3%
	Strongly agree	96	10.7%
Twice-daily brushing	Don't know	498	55.6%
	Strongly disagree	6	0.7%
	Disagree	28	3.1%
	Agree	202	22.6%
	Strongly agree	161	18.0%
importance of regular dental visits	Don't know	509	56.9%
	Strongly disagree	13	1.5%
	Disagree	36	4.0%
	Agree	205	22.9%
	Strongly agree	132	14.7%
Rinsing reduces fluoride effect	Don't know	573	64.0%
	Strongly disagree	56	6.3%
	Disagree	53	5.9%
	Agree	128	14.3%
	Strongly agree	85	9.5%
Maternal Time for Breastfeeding			
Time available for breastfeeding	has adequate time	263	29.39%
	insufficient time	632	70.61%
Household Dietary Sugar Exposure (HDSE)			
child refined carbohydrate intake between meals	Rarely/never (once/week or less)	395	44.1%
	Sometimes (3–4/week)	135	15.1%
	Often/very often (3 + times/day to daily)	365	40.8%
frequency of sugar-containing food consumption	Rarely/never	409	45.7%
	Sometimes	31	3.5%
	Often/very often	455	50.8%
frequency of sugar-containing drink consumption	Rarely/never	618	69.1%
	Sometimes	141	15.8%
	Often/very often	136	15.2%
Early Childhood Caries (ECC)			
dmft score	0 (caries-free)	829	92.6%
	1–3 (mild ECC)	45	5.0%
	≥4 (severe ECC)	21	2.3%
pufa score	0 (no sequelae)	882	98.5%
	1–3 (mild/moderate sequelae)	13	1.5%
Oral Hygiene Status (OHS)			
Plaque score	0 (no visible plaque)	691	77.2%
	1–5 (minimal plaque)	94	10.5%
	6–11 (moderate plaque)	90	10.1%
	12–18 (heavy plaque)	19	2.1%
Oral Hygiene Index (OHI)	0 (excellent)	660	73.7%
	0.1–1.2 (good)	193	21.6%
	1.3–3.0 (fair)	38	4.2%
	> 3.0 (poor)	4	0.4%
Gingival Index	0 (healthy gingiva)	649	72.5%
	1 (mild inflammation)	237	26.5%
	2 (moderate inflammation)	7	0.8%
	3 (severe inflammation)	2	0.2%

Across the six knowledge indicators, the proportion of mothers responding “don't know” ranged from 55.6% (twice-daily brushing) to 77.3% (effectiveness of water fluoridation). Among those expressing a definite opinion, agreement with evidence-based preventive measures varied but remained modest. Household dietary sugar exposure was common. Half of the children (50.8%) consumed sugar-containing foods often or very often, and 40.8% had refined carbohydrate intake between meals at similar frequency. Sugar-containing drink consumption was less frequent, with only 15.2% consuming these often or very often.

Clinically, most children were ECC-free (92.6%). Among those affected, 5.0% had mild ECC (dmft 1–3) and 2.3% had severe ECC (dmft ≥ 4). Pulpal sequelae (pufa score ≥ 1) were present in 1.5% of children. Oral hygiene status was generally favorable. Most children had no visible plaque (77.2%), excellent or good OHI scores (95.3%), and healthy gingiva (72.5%). Mild gingival inflammation was observed in 26.5% of children, while moderate or severe inflammation was rare (<1.0%). Regarding maternal monitoring capacity, most mothers (70.6%) reported insufficient time available for breastfeeding.

### Structural equation model

Five latent constructs met the retention criteria (Table 2). Inter-item correlations ranged from 0.189 to 0.720, with no pair falling below the 0.15 lower bound. For SES, the highest correlation (mother's education × father's education, *ρ* = 0.709) reflected spousal educational homogamy rather than item redundancy. For MOHK and OHS, most correlations exceeded the 0.50 upper bound, consistent with their narrow conceptual domains; no correlation approached the lower bound, and CFA-derived indices confirmed convergent validity (CR = 0.90 and 0.80; AVE = 0.56 for both). Although Cronbach's *α* for OHS was 0.436, retention was supported by the primary homogeneity criterion (minimum *ρ* = 0.491) and satisfactory CR. For HDSE, AVE was 0.36, but CR = 0.59 supported retention. Standardized factor loadings from the DWLS measurement model for all latent constructs are reported in Supplementary Table S1. The model demonstrated acceptable fit: CFI = 0.959, TLI = 0.952, RMSEA = 0.045 [90% CI (0.040, 0.050)], *χ*^2^/df = 2.82. All 1,000 bootstrap resamples converged successfully.

**Table 2:** Inter-item correlations, internal consistency, and convergent validity of reflective constructs,.

Construct	k	*ρ* range (min-max)	*Α*	CR	AVE
SES	4	0.27–0.71^a^	0.73	0.75	0.43
MOHK	6	0.42–0.69^b^	0.88	0.90	0.56
OHS	3	0.49–0.72^b^	0.47	0.80	0.56
ECC	2	0.31^c^	—	—	—
HDSE	3	0.19–0.39	0.56	0.59	0.36^d^

k, number of indicators; ρ, Spearman's correlation; *α*, Cronbach's alpha; CR, composite reliability; AVE, average variance extracted. ^a^Spousal homogamy; ^b^Low *α*, but min *ρ* = 0.491 and CR = 0.80 support retention; ^c^AVE < 0.50 acceptable given CR = 0.59.

Table 3 presents the direct structural path estimates. Socioeconomic status consistently predicted all outcomes: higher SES was associated with greater maternal knowledge (*β* = 0.47), better oral hygiene status (*β* = −0.17), and lower ECC (*β* = −0.15). Oral hygiene status was the strongest proximal predictor of ECC (*β* = 0.32). Child age operated primarily through oral hygiene status (*β* = 0.32), with negligible direct effects on ECC once oral hygiene status was controlled (*β* = 0.07). A knowledge-behavior gap emerged: greater maternal knowledge unexpectedly predicted worse oral hygiene status (*β* = 0.13), evidence that resource constraints limit knowledge translation. Limited maternal time available for breastfeeding directly increased ECC experience (*β* = 0.10). Higher sugar exposure showed a model-significant effect (*β* = 0.13, *p* = 0.039), though the bootstrap interval included zero, requiring cautious interpretation.

**Table 3:** Direct structural path estimates (*N* = 895).

Path	$\beta_{std}$	SE	95% CI	*p*
$\eta_{SES}\;$** →** $\eta_{MOHK}$	+0.467	0.080	[0.407, 0.532]	< 0.001
$\eta_{SES}$** →** $\eta_{OHS}$	−0.171	0.033	[−0.279, −0.047]	0.004
$\eta_{MOHK}$** →** $\eta_{OHS}$	+0.125	0.016	[0.030, 0.209]	0.010
$\chi_{age}\;$** →** $\eta_{OHS}$	+0.317	0.001	[0.263, 0.373]	< 0.001
$\eta_{OHS}$** →** $\eta_{ECC}$	+0.316	0.090	[0.143, 0.414]	0.008
$\eta_{SES}$** →** $\eta_{ECC}$	−0.153	0.064	[−0.259, −0.012]	0.034
$\chi_{bfeeding}$** →** $\eta_{ECC}$	+0.100	0.074	[0.031, 0.171]	0.020
$\eta_{HDSE}$** →** $\eta_{ECC}$	+0.129	0.058	[−0.014, 0.236]	0.084
$\chi_{age}\;$** →** $\eta_{ECC}$	+0.066	0.002	[−0.001, 0.148]	0.056

$\eta_{SES}$, socioeconomic status; $\eta_{MOHK}$, maternal oral health knowledge; $\eta_{OHS}$, oral health status; $\eta_{HDSE}$, household dietary sugar exposure; $\;\chi_{\mathrm{age}}$, child age, $\eta_{ECC},\;\mathrm{early}\;\mathrm{childhood}\;\mathrm{caries}$; $\chi_{\mathrm{breastfeeding}},\;\mathrm{Maternal}\;\mathrm{Time}\;\mathrm{for}\;\mathrm{Breastfeeding}$; $\beta_{std}$, standardised path coefficient; SE, bootstrap standard error; 95% CI, 95% confidence interval from 1,000 bootstrap resamples.

The mediation analysis (Table 4) showed that child age worked entirely through oral hygiene. The indirect path from age to ECC via oral hygiene was 0.10. Socioeconomic status had a total effect of −0.19 on ECC. This came from three parts: a direct path (−0.15), an indirect path through oral hygiene (−0.05), and a smaller indirect path through maternal knowledge (+0.02). All bootstrap confidence intervals excluded zero. SES also had a total effect of −0.11 on oral hygiene. This combined its direct benefit (−0.17) with a small offset through maternal knowledge (+0.06). The total effect of SES on ECC (−0.19) was larger than any single direct path. This highlights how socioeconomic disadvantage accumulates across multiple pathways to increase ECC risk in this population.

**Table 4:** Significant indirect (mediated) path effects.

Indirect pathway	$\beta_{std}$.	95% CI
$\eta_{SES}\;$** →** $\eta_{MOHK}$** →** $\eta_{OHS}$	+0.058	[0.012, 0.111]
$\eta_{SES}\;$** →** $\eta_{OHS}$** →** $\eta_{ECC}$	−0.054	[−0.090, −0.010]
$\eta_{SES}\;$** →** $\eta_{MOHK}$** →** $\eta_{OHS}$** →** $\eta_{ECC}$	+0.018	[0.003, 0.035]
$\eta_{MOHK}$** →** $\eta_{OHS}$** →** $\eta_{ECC}$	+0.039	[0.006, 0.072]
$\chi_{\mathrm{age}}$** →** $\eta_{OHS}$**→** $\eta_{ECC}$	+0.100	[0.042, 0.138]

$\eta_{SES}$, socioeconomic status; $\eta_{MOHK}$, maternal oral health knowledge; $\eta_{OHS}$, oral health status; $\;\chi_{\mathrm{age}}$ child age, $\eta_{ECC},\;\mathrm{early}\;\mathrm{childhood}\;\mathrm{caries}$; $\beta_{std}$, standardised path coefficient; 95% CI, 95% confidence interval from 1,000 bootstrap resamples.

**Figure 2:**
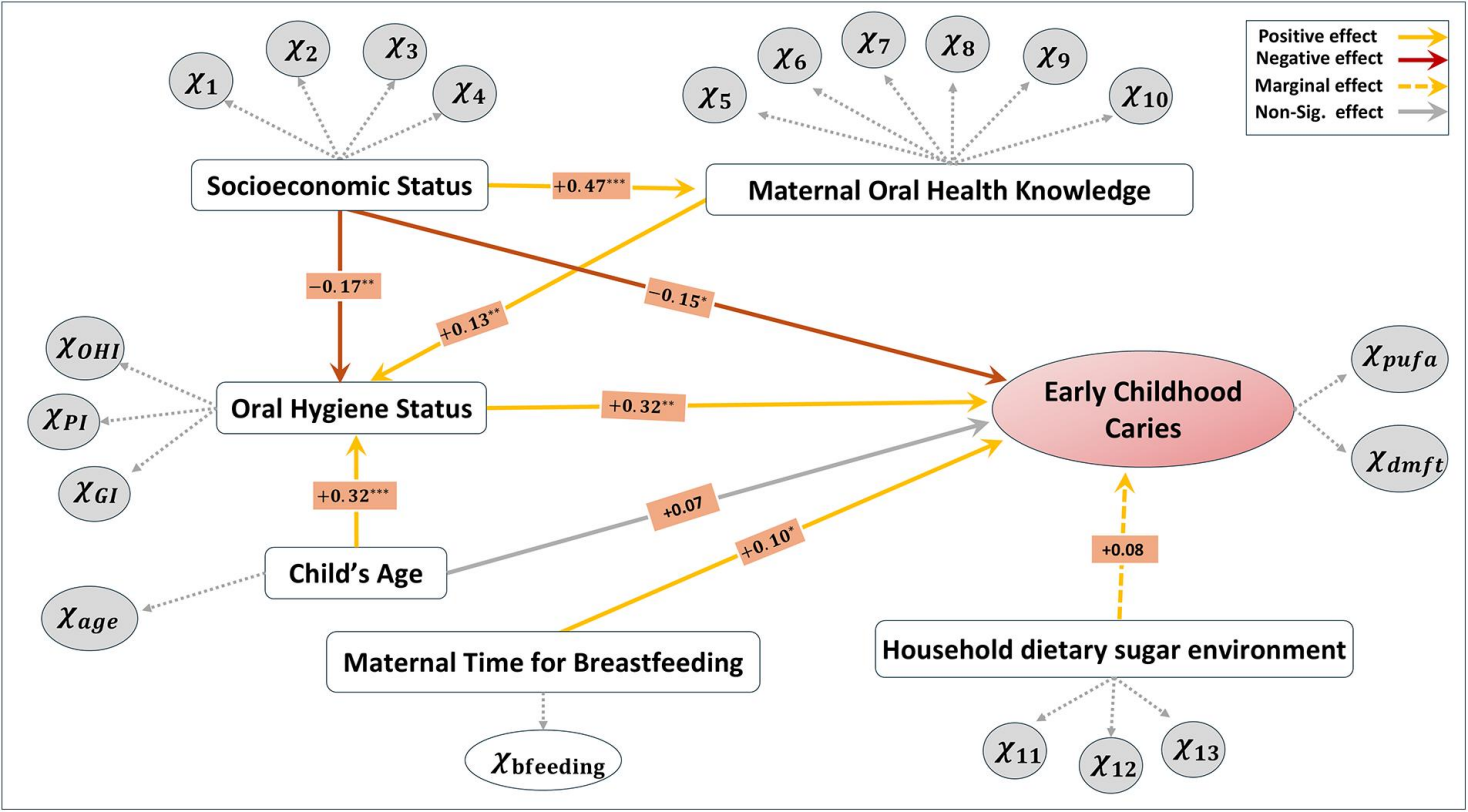
Structural equation model showing standardized direct and indirect pathways associated with early childhood caries.

Figure 2 illustrates the standardized direct path coefficients and total effects estimated from the DWLS-SEM. Orange arrows indicate positive associations, red arrows negative associations, orange dashed arrows marginal associations, and grey arrows non-significant paths. Significance levels: *** *p* < 0.001, ** *p* < 0.01, * *p* < 0.05.

## Discussion

This cross-sectional study examined physical and social determinants of ECC among children aged 0–60 months in Ile-Ife Central Local Government, Nigeria, using DWLS-SEM. The results showed that SES was the most important distal factor. It showed both direct and indirect protective associations with ECC through two distinct patterns: a resource-access pathway via oral hygiene status, and a knowledge-mediated pathway via Maternal oral health knowledge. Oral hygiene status, which was worse among older children, showed the strongest proximal association with ECC. A counterintuitive finding emerged: higher maternal knowledge was associated with worse oral hygiene scores once SES was held constant. This suggests a gap between knowledge and behavior, which is consistent with the CSDH framework, indicating that structural and contextual constraints may prevent knowledge from being translated into effective oral health practices.

This is the first study **applying SEM to ECC risk factors** in Nigeria and in Africa, one of the few studies using SEM analysis to identify the risk factors for ECC in a population (33, 58, 59). One of the strengths of the current study is that it offers valuable insights into the physical and social risk indicators for ECC among the study population through comprehensive data collection and advanced analytical methods.

While the study has notable strengths, its cross-sectional design limits causal inference, and reliance on self-reported data introduces potential recall and social desirability biases. The study was conducted within a single suburban local government area with a relatively educated caregiver population (more than 85% with secondary or tertiary education), which may limit generalizability to rural Nigerian communities, northern regions, or other African settings with different socioeconomic conditions, dietary patterns, and fluoride exposure. Additionally, the exclusion of key variables such as genetic predisposition and access to dental care may have resulted in residual confounding. The relatively low ECC prevalence (7.4%) compared to the complexity of the modeled pathways warrants cautious interpretation, as parameter estimates for distal multi-step associations may be imprecise and require replication in populations with higher disease burden. Despite these limitations, the current study lays a strong foundation for designing culturally appropriate, targeted interventions to address ECC in the study population and other populations with a similar epidemiological profile.

First, the study findings showed a positive association between household dietary sugar exposure and ECC. This aligns with existing evidence linking sugar consumption to dental caries in young children (60). Although the bootstrap confidence interval for this pathway marginally crossed zero, this likely reflects the low ECC prevalence in this sample (7.4%) limiting statistical power for detecting modest dietary associations. The finding is consistent with established literature showing that the quantity, frequency, and timing of sugar intake from foods and sugar-sweetened beverages are each associated with dental caries risk (61, 62).

Poor oral hygiene practices, such as irregular brushing, may further strengthen this association (63). The household dietary environment is shaped by maternal knowledge, food accessibility, and cultural practices. Prior studies have shown that maternal caries status and salivary S. mutans levels are strongly correlated with child outcomes (45, 64–66). This highlights the value of integrated maternal and child oral healthcare. Addressing dietary sugar practices at the household level is therefore an important target for ECC prevention (67). However, reducing ECC burden requires strategies that extend beyond individual households. Public health policies—including sugar taxes, restrictions on marketing sugary foods to children, and parental education about hidden sugars in processed foods and beverages—may help reduce population-level sugar consumption (68). Programs that provide maternal oral health screenings, treatment, and education during pregnancy have also been associated with lower child ECC risk (69). A holistic approach addressing multiple social and physical determinants across macro, meso, exo, and micro levels is needed to achieve meaningful reductions in ECC burden (70). Second, the study findings highlight the need to promote consistent oral care for children.

Second, oral hygiene status showed the strongest proximal association with ECC among all variables examined. This finding aligns with previous Nigerian studies that identified poor oral hygiene as a key correlate of dental caries in young children (32, 46, 47). Older children in this sample had poorer OHS. This pattern is consistent with greater plaque accumulation over time. The indirect pathway from age to ECC through OHS was significant, while the direct association between age and ECC was not. This suggests that age is related to dental caries primarily through its association with plaque accumulation rather than through any independent age-related pathway. Effective plaque control depends on brushing frequency and proper tools (65). Establishing twice-daily brushing routines early in life is therefore important (71). The findings highlight the need for improved supervision and routine oral hygiene practices for children (71–74). Strategies that support parents in establishing consistent oral care routines may be particularly beneficial for reducing ECC risk in this population.

Third, MOHK showed a socially patterned association with socioeconomic status. Higher SES was associated with greater knowledge. However, a counterintuitive finding emerged: once SES was held constant, higher knowledge was associated with worse OHS score. This knowledge-behavior gap is consistent with the CSDH framework, where structural resource constraints and competing household priorities may override cognitive awareness when socioeconomic disadvantage is not addressed (75). Previous research in this study population identified an association between maternal knowledge and ECC (76). The current findings reaffirm the importance of this construct using a more rigorous analytical approach. Despite the observed gap, maternal knowledge showed a significant indirect protective association with ECC through OHS. These supports integrating maternal oral health education into child healthcare programs. However, educational strategies must be accompanied by structural measures that enable families to act on acquired knowledge (75, 77).

Fourth, the resource-access channel was the dominant pathway linking socioeconomic status to lower ECC risk. This means that families with higher SES had better access to oral hygiene resources, which in turn was associated with fewer ECC. The knowledge pathway played only a small role. The Biopsychosocial Model suggests that poor oral hygiene and high sugar consumption are influenced by cultural norms (78–80). Therefore, culturally sensitive education programs tailored to local customs may be needed. Population-level policies, such as sugar taxes and restrictions on marketing sugary foods to children, are also important. Community-based interventions that offer free or low-cost dental care and education may help bridge the gap between knowledge and action by reducing practical barriers (81, 82).

Fifth, several non-significant findings were theoretically informative. The direct association between the child's age and ECC was non-significant once oral hygiene status was included in the model. This supports the interpretation that age is related to dental caries primarily through plaque accumulation rather than through any independent pathway. The direct association between SES and ECC, while significant, captured only part of the total SES-ECC relationship. The total association substantially exceeded the direct coefficient alone. Analyses restricted to direct paths would therefore underestimate the socioeconomic gradient in ECC. The absence of a significant direct path from maternal knowledge to ECC (knowledge was associated only indirectly through oral hygiene) suggests that cognitive awareness alone shows no independent association with dental caries outcomes when structural mediators are included in the model.

These findings have implications for policy, practice, and research. The identification of oral hygiene status, socioeconomic status, and dietary sugar exposure as key correlates highlights several needs. These include integrating oral health into child health policies, promoting integrated maternal and child oral healthcare, advocating for universal health coverage including dental care, and implementing public health measures to reduce dietary ECC risk. Addressing ECC requires collaborative efforts among policymakers, healthcare providers, educators, and communities. Culturally appropriate, evidence-based interventions that address the multifactorial nature of ECC are needed.

## Conclusion

This study confirms that early childhood caries in Ile-Ife, Nigeria is structurally determined, not simply a matter of maternal knowledge. Socioeconomic status consistently predicted better oral hygiene and lower ECC risk, and this effect operated through resource access rather than through knowledge alone. The knowledge-behavior gap was clear: mothers who were knowledgeable but lived in disadvantaged circumstances could not translate what they knew into better oral hygiene for their children. The implication is direct; educational interventions alone will fail. What is needed is a three-pronged response: clinical promotion of early plaque control, maternal oral health education embedded within routine child healthcare, and upstream policies that reduce sugar exposure and expand access to preventive dental care. Without addressing these structural barriers, the socioeconomic gradient in childhood caries will persist.

## Data Availability

The datasets presented in this study can be found in online repositories. The names of the repository/repositories and accession number(s) can be found below: The data is available online. Data link: 10.6084/m9.figshare.28544336.
