# Supplementary material for: Structural equation modeling to identify social and physical risk indicators of early childhood caries in suburban Nigerian population
Source: Front Oral Health. 2026 Jul 20;7:1846301. doi: 10.3389/froh.2026.1846301 (PMC13429854; doi:10.3389/froh.2026.1846301)
Supplement: Supplementary file 1 [file Table1.docx]

**Supplementary Table 1.** Factor Loadings of Latent Constructs

| **Construct / Indicator** | **λ (std)** | **SE** | **z** | **p** |
| --- | --- | --- | --- | --- |
| **SES \| CR = 0.75, AVE = 0.43** | | | | |
| Mother's education (reference) | 0.87 | - | - | - |
| Father's education | 0.84 | 0.04 | 21.94 | < 0.001 |
| Mother's occupation | 0.41 | 0.04 | 11.52 | < 0.001 |
| Father's occupation | 0.42 | 0.03 | 11.95 | < 0.001 |
| **MOHK \| CR = 0.90, AVE = 0.56** | | | | |
| Fluoride toothpaste effectiveness (reference) | 0.76 | - | - | - |
| Fluoride more important than brushing | 0.80 | 0.07 | 19.56 | < 0.001 |
| Twice-daily brushing with fluoride toothpaste | 0.82 | 0.08 | 19.90 | < 0.001 |
| Importance of regular dental check-ups | 0.74 | 0.07 | 18.43 | < 0.001 |
| Rinsing after brushing reduces fluoride effect | 0.73 | 0.07 | 18.11 | < 0.001 |
| Effectiveness of water fluoridation | 0.73 | 0.07 | 18.28 | < 0.001 |
| **OHS \| CR = 0.80, AVE = 0.56** | | | | |
| Oral Hygiene Index | 0.84 | - | - | - |
| Gingival Index (GI) | 0.80 | 0.05 | 18.74 | < 0.001 |
| Plaque score | 0.64 | 0.05 | 15.12 | < 0.001 |
| **ECC \| bivariate ρ (dmft × pufa) = 0.311, p < 0.001** | | | | |
| dmft score (reference) | 0.85 | - | - | - |
| pufa score | 0.37 | 0.04 | 9.14 | < 0.001 |
| **HDSE \| CR = 0.59, AVE = 0.36** | | | | |
| Refined carbohydrate intake between meals (reference) | 0.75 | - | - | - |
| Sugar-containing food consumption frequency | 0.70 | 0.06 | 16.35 | < 0.001 |
| Sugar-containing drink consumption frequency | 0.37 | 0.05 | 9.78 | < 0.001 |
